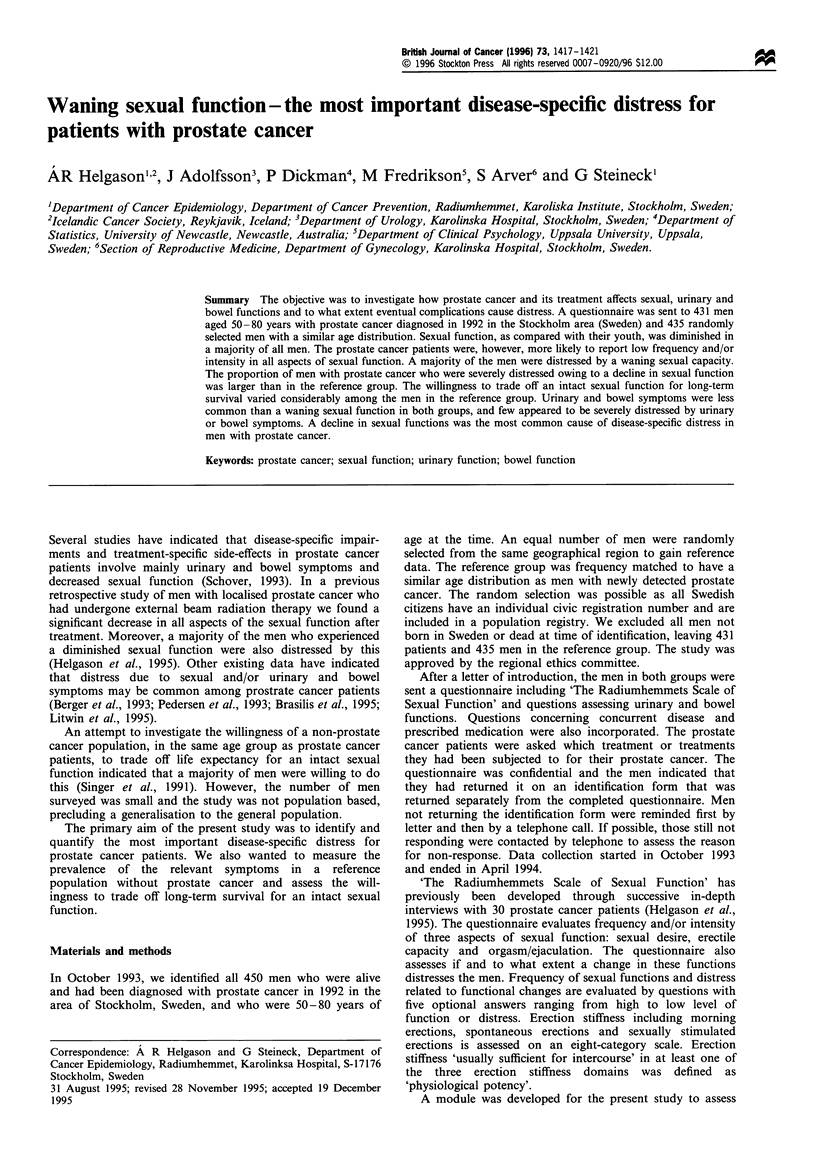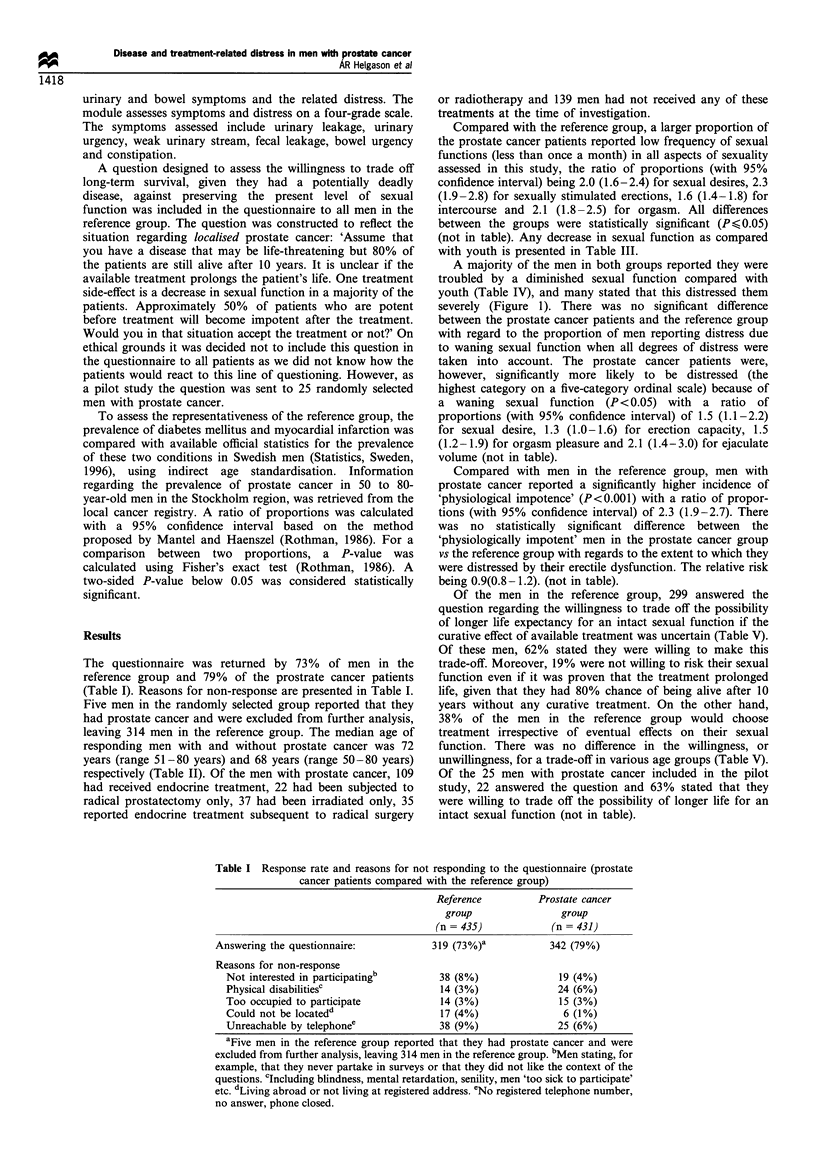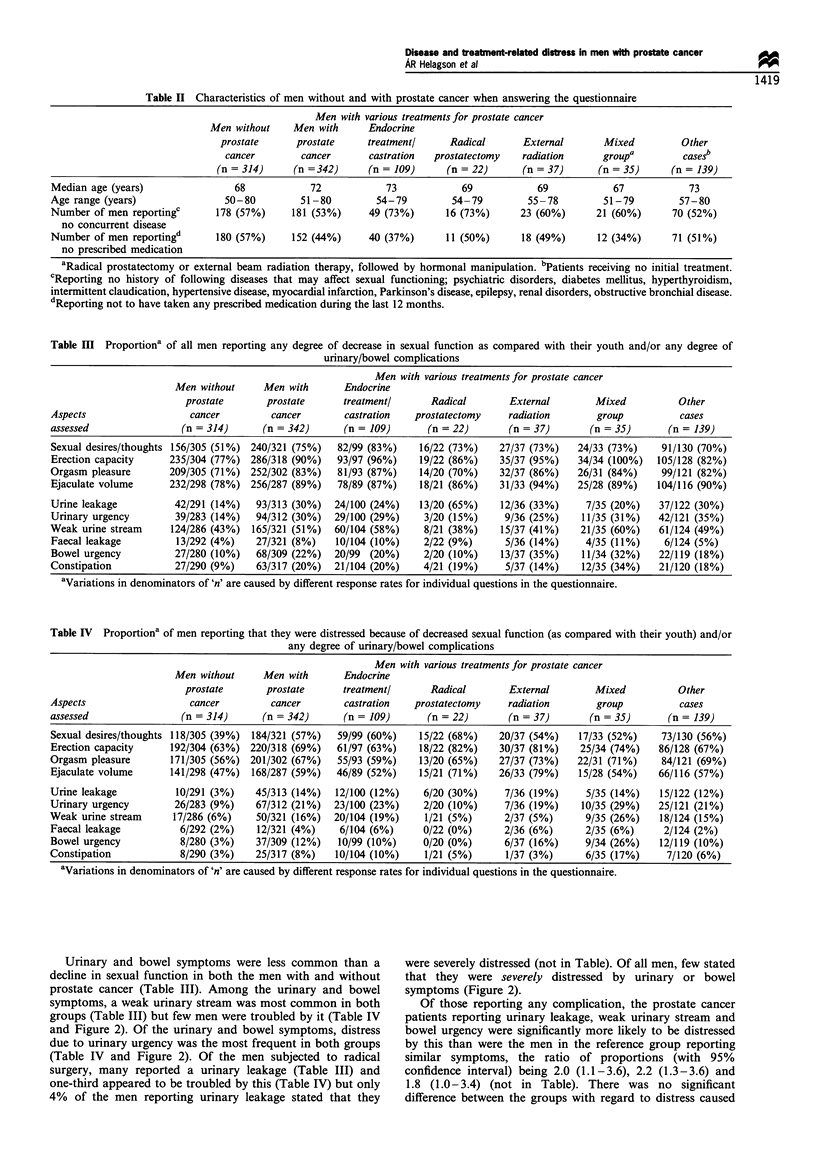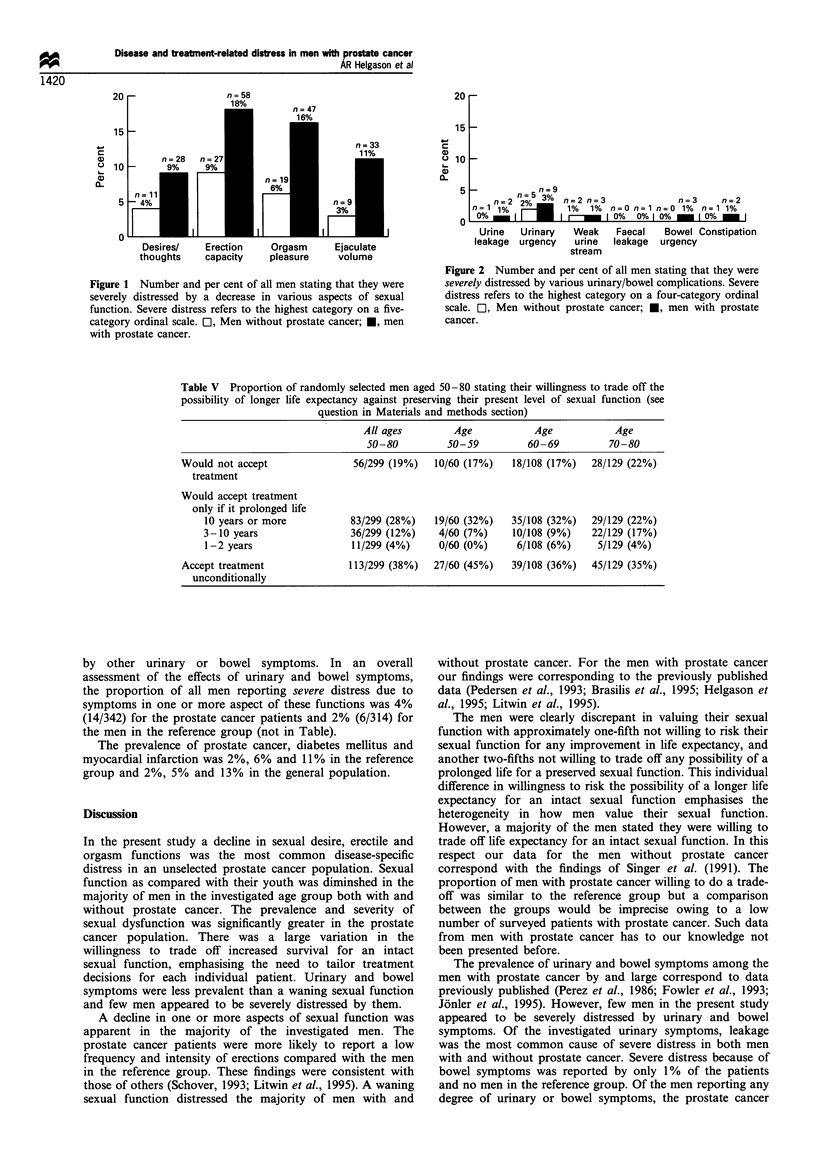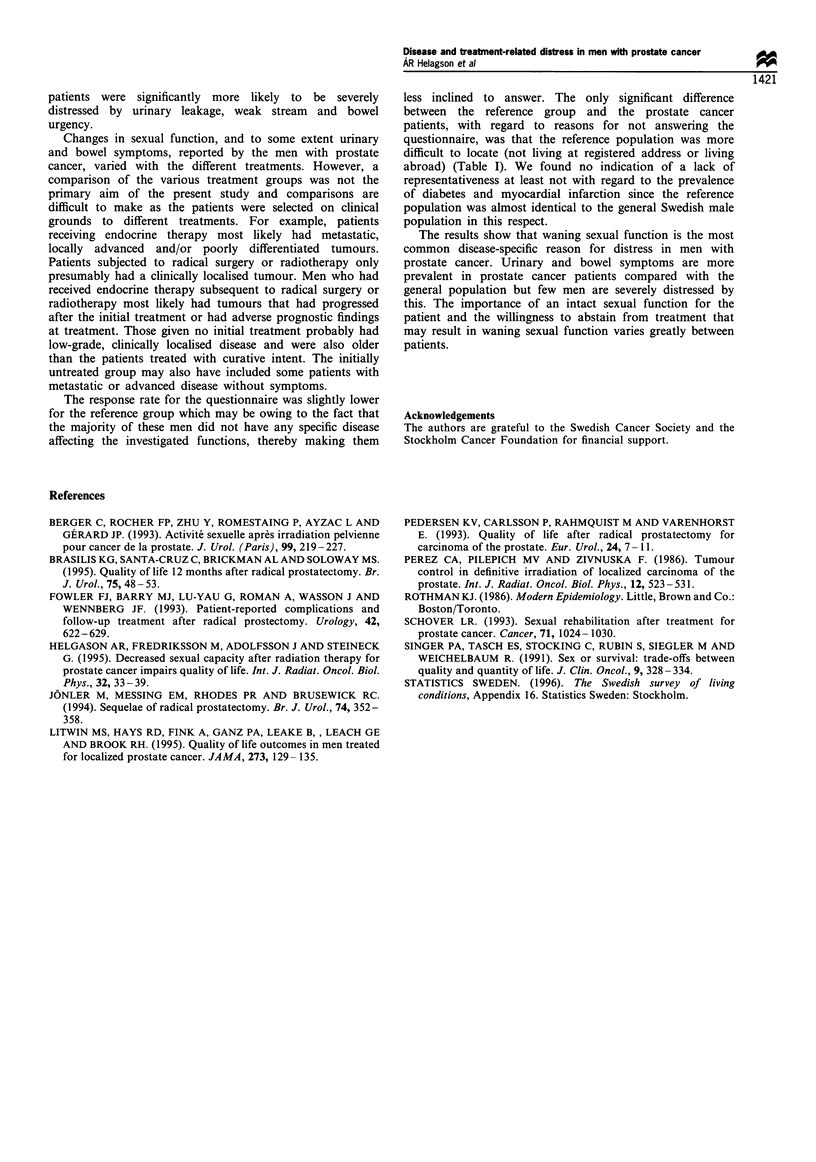# Waning sexual function--the most important disease-specific distress for patients with prostate cancer.

**DOI:** 10.1038/bjc.1996.268

**Published:** 1996-06

**Authors:** A. R. Helgason, J. Adolfsson, P. Dickman, M. Fredrikson, S. Arver, G. Steineck

**Affiliations:** Department of Cancer Epidemiology, Radiumhemmet, Karolinska Institute, Stockholm, Sweden.

## Abstract

The objective was to investigate how prostate cancer and its treatment affects sexual, urinary and bowel functions and to what extent eventual complications cause distress. A questionnaire was sent to 431 men aged 50-80 years with prostate cancer diagnosed in 1992 in the Stockholm area (Sweden) and 435 randomly selected men with a similar age distribution. Sexual function, as compared with their youth, was diminished in a majority of all men. The prostate cancer patients were, however, more likely to report low frequency and/or intensity in all aspects of sexual function. A majority of the men were distressed by a waning sexual capacity. The proportion of men with prostate cancer who were severely distressed owing to a decline in sexual function was larger than in the reference group. The willingness to trade off an intact sexual function for long-term survival varied considerably among the men in the reference group. Urinary and bowel symptoms were less common than a waning sexual function in both groups, and few appeared to be severely distressed by urinary or bowel symptoms. A decline in sexual functions was the most common cause of disease-specific distress in men with prostate cancer.


British Journal of Cancer (1996) 73, 1417-1421
© 1996 Stockton Press All rights reserved 0007-0920/96 $12.00

### Waning sexual function – the most important disease-specific distress for patients with prostate cancer

ÁR Helgason[1,2], J Adolfsson[3], P Dickman[4], M Fredrikson[5], S Arver[6] and G Steineck[1]

[1]*Department of Cancer Epidemiology, Department of Cancer Prevention, Radiumhemmet, Karoliska Institute, Stockholm, Sweden;* [2]*Icelandic Cancer Society, Reykjavik, Iceland;* [3]*Department of Urology, Karolinska Hospital, Stockholm, Sweden;* [4]*Department of Statistics, University of Newcastle, Newcastle, Australia;* [5]*Department of Clinical Psychology, Uppsala University, Uppsala, Sweden;* [6]*Section of Reproductive Medicine, Department of Gynecology, Karolinska Hospital, Stockholm, Sweden.*

**Summary** The objective was to investigate how prostate cancer and its treatment affects sexual, urinary and bowel functions and to what extent eventual complications cause distress. A questionnaire was sent to 431 men aged 50–80 years with prostate cancer diagnosed in 1992 in the Stockholm area (Sweden) and 435 randomly selected men with a similar age distribution. Sexual function, as compared with their youth, was diminished in a majority of all men. The prostate cancer patients were, however, more likely to report low frequency and/or intensity in all aspects of sexual function. A majority of the men were distressed by a waning sexual capacity. The proportion of men with prostate cancer who were severely distressed owing to a decline in sexual function was larger than in the reference group. The willingness to trade off an intact sexual function for long-term survival varied considerably among the men in the reference group. Urinary and bowel symptoms were less common than a waning sexual function in both groups, and few appeared to be severely distressed by urinary or bowel symptoms. A decline in sexual functions was the most common cause of disease-specific distress in men with prostate cancer.

**Keywords:** prostate cancer; sexual function; urinary function; bowel function

Several studies have indicated that disease-specific impairments and treatment-specific side-effects in prostate cancer patients involve mainly urinary and bowel symptoms and decreased sexual function (Schover, 1993). In a previous retrospective study of men with localised prostate cancer who had undergone external beam radiation therapy we found a significant decrease in all aspects of the sexual function after treatment. Moreover, a majority of the men who experienced a diminished sexual function were also distressed by this (Helgason *et al.*, 1995). Other existing data have indicated that distress due to sexual and/or urinary and bowel symptoms may be common among prostate cancer patients (Berger *et al.*, 1993; Pedersen *et al.*, 1993; Brasilis *et al.*, 1995; Litwin *et al.*, 1995).

An attempt to investigate the willingness of a non-prostate cancer population, in the same age group as prostate cancer patients, to trade off life expectancy for an intact sexual function indicated that a majority of men were willing to do this (Singer *et al.*, 1991). However, the number of men surveyed was small and the study was not population based, precluding a generalisation to the general population.

The primary aim of the present study was to identify and quantify the most important disease-specific distress for prostate cancer patients. We also wanted to measure the prevalence of the relevant symptoms in a reference population without prostate cancer and assess the willingness to trade off long-term survival for an intact sexual function.

### Materials and methods

In October 1993, we identified all 450 men who were alive and had been diagnosed with prostate cancer in 1992 in the area of Stockholm, Sweden, and who were 50–80 years of age at the time. An equal number of men were randomly selected from the same geographical region to gain reference data. The reference group was frequency matched to have a similar age distribution as men with newly detected prostate cancer. The random selection was possible as all Swedish citizens have an individual civic registration number and are included in a population registry. We excluded all men not born in Sweden or dead at time of identification, leaving 431 patients and 435 men in the reference group. The study was approved by the regional ethics committee.

After a letter of introduction, the men in both groups were sent a questionnaire including 'The Radiumhemmets Scale of Sexual Function' and questions assessing urinary and bowel functions. Questions concerning concurrent disease and prescribed medication were also incorporated. The prostate cancer patients were asked which treatment or treatments they had been subjected to for their prostate cancer. The questionnaire was confidential and the men indicated that they had returned it on an identification form that was returned separately from the completed questionnaire. Men not returning the identification form were reminded first by letter and then by a telephone call. If possible, those still not responding were contacted by telephone to assess the reason for non-response. Data collection started in October 1993 and ended in April 1994.

'The Radiumhemmets Scale of Sexual Function' has previously been developed through successive in-depth interviews with 30 prostate cancer patients (Helgason *et al.*, 1995). The questionnaire evaluates frequency and/or intensity of three aspects of sexual function: sexual desire, erectile capacity and orgasm/ejaculation. The questionnaire also assesses if and to what extent a change in these functions distresses the men. Frequency of sexual functions and distress related to functional changes are evaluated by questions with five optional answers ranging from high to low level of function or distress. Erection stiffness including morning erections, spontaneous erections and sexually stimulated erections is assessed on an eight-category scale. Erection stiffness 'usually sufficient for intercourse' in at least one of the three erection stiffness domains was defined as 'physiological potency'.

A module was developed for the present study to assess

Correspondence: Á R Helgason and G Steineck, Department of Cancer Epidemiology, Radiumhemmet, Karolinksa Hospital, S-17176 Stockholm, Sweden
31 August 1995; revised 28 November 1995; accepted 19 December 1995

urinary and bowel symptoms and the related distress. The module assesses symptoms and distress on a four-grade scale. The symptoms assessed include urinary leakage, urinary urgency, weak urinary stream, fecal leakage, bowel urgency and constipation.

A question designed to assess the willingness to trade off long-term survival, given they had a potentially deadly disease, against preserving the present level of sexual function was included in the questionnaire to all men in the reference group. The question was constructed to reflect the situation regarding *localised* prostate cancer: 'Assume that you have a disease that may be life-threatening but 80% of the patients are still alive after 10 years. It is unclear if the available treatment prolongs the patient's life. One treatment side-effect is a decrease in sexual function in a majority of the patients. Approximately 50% of patients who are potent before treatment will become impotent after the treatment. Would you in that situation accept the treatment or not?' On ethical grounds it was decided not to include this question in the questionnaire to all patients as we did not know how the patients would react to this line of questioning. However, as a pilot study the question was sent to 25 randomly selected men with prostate cancer.

To assess the representativeness of the reference group, the prevalence of diabetes mellitus and myocardial infarction was compared with available official statistics for the prevalence of these two conditions in Swedish men (Statistics, Sweden, 1996), using indirect age standardisation. Information regarding the prevalence of prostate cancer in 50 to 80-year-old men in the Stockholm region, was retrieved from the local cancer registry. A ratio of proportions was calculated with a 95% confidence interval based on the method proposed by Mantel and Haenszel (Rothman, 1986). For a comparison between two proportions, a $P$-value was calculated using Fisher's exact test (Rothman, 1986). A two-sided $P$-value below 0.05 was considered statistically significant.

### Results

The questionnaire was returned by 73% of men in the reference group and 79% of the prostrate cancer patients (Table I). Reasons for non-response are presented in Table I. Five men in the randomly selected group reported that they had prostate cancer and were excluded from further analysis, leaving 314 men in the reference group. The median age of responding men with and without prostate cancer was 72 years (range 51–80 years) and 68 years (range 50–80 years) respectively (Table II). Of the men with prostate cancer, 109 had received endocrine treatment, 22 had been subjected to radical prostatectomy only, 37 had been irradiated only, 35 reported endocrine treatment subsequent to radical surgery or radiotherapy and 139 men had not received any of these treatments at the time of investigation.

Compared with the reference group, a larger proportion of the prostate cancer patients reported low frequency of sexual functions (less than once a month) in all aspects of sexuality assessed in this study, the ratio of proportions (with 95% confidence interval) being 2.0 (1.6–2.4) for sexual desires, 2.3 (1.9–2.8) for sexually stimulated erections, 1.6 (1.4–1.8) for intercourse and 2.1 (1.8–2.5) for orgasm. All differences between the groups were statistically significant ($P \leqslant 0.05$) (not in table). Any decrease in sexual function as compared with youth is presented in Table III.

A majority of the men in both groups reported they were troubled by a diminished sexual function compared with youth (Table IV), and many stated that this distressed them severely (Figure 1). There was no significant difference between the prostate cancer patients and the reference group with regard to the proportion of men reporting distress due to waning sexual function when all degrees of distress were taken into account. The prostate cancer patients were, however, significantly more likely to be distressed (the highest category on a five-category ordinal scale) because of a waning sexual function ($P < 0.05$) with a ratio of proportions (with 95% confidence interval) of 1.5 (1.1–2.2) for sexual desire, 1.3 (1.0–1.6) for erection capacity, 1.5 (1.2–1.9) for orgasm pleasure and 2.1 (1.4–3.0) for ejaculate volume (not in table).

Compared with men in the reference group, men with prostate cancer reported a significantly higher incidence of 'physiological impotence' ($P < 0.001$) with a ratio of proportions (with 95% confidence interval) of 2.3 (1.9–2.7). There was no statistically significant difference between the 'physiologically impotent' men in the prostate cancer group *vs* the reference group with regards to the extent to which they were distressed by their erectile dysfunction. The relative risk being 0.9(0.8–1.2). (not in table).

Of the men in the reference group, 299 answered the question regarding the willingness to trade off the possibility of longer life expectancy for an intact sexual function if the curative effect of available treatment was uncertain (Table V). Of these men, 62% stated they were willing to make this trade-off. Moreover, 19% were not willing to risk their sexual function even if it was proven that the treatment prolonged life, given that they had 80% chance of being alive after 10 years without any curative treatment. On the other hand, 38% of the men in the reference group would choose treatment irrespective of eventual effects on their sexual function. There was no difference in the willingness, or unwillingness, for a trade-off in various age groups (Table V). Of the 25 men with prostate cancer included in the pilot study, 22 answered the question and 63% stated that they were willing to trade off the possibility of longer life for an intact sexual function (not in table).

**Table I** Response rate and reasons for not responding to the questionnaire (prostate cancer patients compared with the reference group)

| | *Reference group* (n = *435*) | *Prostate cancer group* (n = *431*) |
|---|---|---|
| Answering the questionnaire: | 319 (73%)[a] | 342 (79%) |
| Reasons for non-response | | |
| Not interested in participating[b] | 38 (8%) | 19 (4%) |
| Physical disabilities[c] | 14 (3%) | 24 (6%) |
| Too occupied to participate | 14 (3%) | 15 (3%) |
| Could not be located[d] | 17 (4%) | 6 (1%) |
| Unreachable by telephone[e] | 38 (9%) | 25 (6%) |

[a]Five men in the reference group reported that they had prostate cancer and were excluded from further analysis, leaving 314 men in the reference group. [b]Men stating, for example, that they never partake in surveys or that they did not like the context of the questions. [c]Including blindness, mental retardation, senility, men 'too sick to participate' etc. [d]Living abroad or not living at registered address. [e]No registered telephone number, no answer, phone closed.

**Table II** Characteristics of men without and with prostate cancer when answering the questionnaire

| | *Men without prostate cancer* (n = *314*) | *Men with prostate cancer* (n = *342*) | *Men with various treatments for prostate cancer* — *Endocrine treatment/ castration* (n = *109*) | *Radical prostatectomy* (n = *22*) | *External radiation* (n = *37*) | *Mixed group*[a] (n = *35*) | *Other cases*[b] (n = *139*) |
|---|---|---|---|---|---|---|---|
| Median age (years) | 68 | 72 | 73 | 69 | 69 | 67 | 73 |
| Age range (years) | 50-80 | 51-80 | 54-79 | 54-79 | 55-78 | 51-79 | 57-80 |
| Number of men reporting[c] no concurrent disease | 178 (57%) | 181 (53%) | 49 (73%) | 16 (73%) | 23 (60%) | 21 (60%) | 70 (52%) |
| Number of men reporting[d] no prescribed medication | 180 (57%) | 152 (44%) | 40 (37%) | 11 (50%) | 18 (49%) | 12 (34%) | 71 (51%) |

[a]Radical prostatectomy or external beam radiation therapy, followed by hormonal manipulation. [b]Patients receiving no initial treatment. [c]Reporting no history of following diseases that may affect sexual functioning; psychiatric disorders, diabetes mellitus, hyperthyroidism, intermittent claudication, hypertensive disease, myocardial infarction, Parkinson's disease, epilepsy, renal disorders, obstructive bronchial disease. [d]Reporting not to have taken any prescribed medication during the last 12 months.

**Table III** Proportion[a] of all men reporting any degree of decrease in sexual function as compared with their youth and/or any degree of urinary/bowel complications

| *Aspects assessed* | *Men without prostate cancer* (n = *314*) | *Men with prostate cancer* (n = *342*) | *Men with various treatments for prostate cancer* — *Endocrine treatment/ castration* (n = *109*) | *Radical prostatectomy* (n = *22*) | *External radiation* (n = *37*) | *Mixed group* (n = *35*) | *Other cases* (n = *139*) |
|---|---|---|---|---|---|---|---|
| Sexual desires/thoughts | 156/305 (51%) | 240/321 (75%) | 82/99 (83%) | 16/22 (73%) | 27/37 (73%) | 24/33 (73%) | 91/130 (70%) |
| Erection capacity | 235/304 (77%) | 286/318 (90%) | 93/97 (96%) | 19/22 (86%) | 35/37 (95%) | 34/34 (100%) | 105/128 (82%) |
| Orgasm pleasure | 209/305 (71%) | 252/302 (83%) | 81/93 (87%) | 14/20 (70%) | 32/37 (86%) | 26/31 (84%) | 99/121 (82%) |
| Ejaculate volume | 232/298 (78%) | 256/287 (89%) | 78/89 (87%) | 18/21 (86%) | 31/33 (94%) | 25/28 (89%) | 104/116 (90%) |
| Urine leakage | 42/291 (14%) | 93/313 (30%) | 24/100 (24%) | 13/20 (65%) | 12/36 (33%) | 7/35 (20%) | 37/122 (30%) |
| Urinary urgency | 39/283 (14%) | 94/312 (30%) | 29/100 (29%) | 3/20 (15%) | 9/36 (25%) | 11/35 (31%) | 42/121 (35%) |
| Weak urine stream | 124/286 (43%) | 165/321 (51%) | 60/104 (58%) | 8/21 (38%) | 15/37 (41%) | 21/35 (60%) | 61/124 (49%) |
| Faecal leakage | 13/292 (4%) | 27/321 (8%) | 10/104 (10%) | 2/22 (9%) | 5/36 (14%) | 4/35 (11%) | 6/124 (5%) |
| Bowel urgency | 27/280 (10%) | 68/309 (22%) | 20/99 (20%) | 2/20 (10%) | 13/37 (35%) | 11/34 (32%) | 22/119 (18%) |
| Constipation | 27/290 (9%) | 63/317 (20%) | 21/104 (20%) | 4/21 (19%) | 5/37 (14%) | 12/35 (34%) | 21/120 (18%) |

[a]Variations in denominators of '*n*' are caused by different response rates for individual questions in the questionnaire.

**Table IV** Proportion[a] of men reporting that they were distressed because of decreased sexual function (as compared with their youth) and/or any degree of urinary/bowel complications

| *Aspects assessed* | *Men without prostate cancer* (n = *314*) | *Men with prostate cancer* (n = *342*) | *Men with various treatments for prostate cancer* — *Endocrine treatment/ castration* (n = *109*) | *Radical prostatectomy* (n = *22*) | *External radiation* (n = *37*) | *Mixed group* (n = *35*) | *Other cases* (n = *139*) |
|---|---|---|---|---|---|---|---|
| Sexual desires/thoughts | 118/305 (39%) | 184/321 (57%) | 59/99 (60%) | 15/22 (68%) | 20/37 (54%) | 17/33 (52%) | 73/130 (56%) |
| Erection capacity | 192/304 (63%) | 220/318 (69%) | 61/97 (63%) | 18/22 (82%) | 30/37 (81%) | 25/34 (74%) | 86/128 (67%) |
| Orgasm pleasure | 171/305 (56%) | 201/302 (67%) | 55/93 (59%) | 13/20 (65%) | 27/37 (73%) | 22/31 (71%) | 84/121 (69%) |
| Ejaculate volume | 141/298 (47%) | 168/287 (59%) | 46/89 (52%) | 15/21 (71%) | 26/33 (79%) | 15/28 (54%) | 66/116 (57%) |
| Urine leakage | 10/291 (3%) | 45/313 (14%) | 12/100 (12%) | 6/20 (30%) | 7/36 (19%) | 5/35 (14%) | 15/122 (12%) |
| Urinary urgency | 26/283 (9%) | 67/312 (21%) | 23/100 (23%) | 2/20 (10%) | 7/36 (19%) | 10/35 (29%) | 25/121 (21%) |
| Weak urine stream | 17/286 (6%) | 50/321 (16%) | 20/104 (19%) | 1/21 (5%) | 2/37 (5%) | 9/35 (26%) | 18/124 (15%) |
| Faecal leakage | 6/292 (2%) | 12/321 (4%) | 6/104 (6%) | 0/22 (0%) | 2/36 (6%) | 2/35 (6%) | 2/124 (2%) |
| Bowel urgency | 8/280 (3%) | 37/309 (12%) | 10/99 (10%) | 0/20 (0%) | 6/37 (16%) | 9/34 (26%) | 12/119 (10%) |
| Constipation | 8/290 (3%) | 25/317 (8%) | 10/104 (10%) | 1/21 (5%) | 1/37 (3%) | 6/35 (17%) | 7/120 (6%) |

[a]Variations in denominators of '*n*' are caused by different response rates for individual questions in the questionnaire.

Urinary and bowel symptoms were less common than a decline in sexual function in both the men with and without prostate cancer (Table III). Among the urinary and bowel symptoms, a weak urinary stream was most common in both groups (Table III) but few men were troubled by it (Table IV and Figure 2). Of the urinary and bowel symptoms, distress due to urinary urgency was the most frequent in both groups (Table IV and Figure 2). Of the men subjected to radical surgery, many reported a urinary leakage (Table III) and one-third appeared to be troubled by this (Table IV) but only 4% of the men reporting urinary leakage stated that they were severely distressed (not in Table). Of all men, few stated that they were *severely* distressed by urinary or bowel symptoms (Figure 2).

Of those reporting any complication, the prostate cancer patients reporting urinary leakage, weak urinary stream and bowel urgency were significantly more likely to be distressed by this than were the men in the reference group reporting similar symptoms, the ratio of proportions (with 95% confidence interval) being 2.0 (1.1-3.6), 2.2 (1.3-3.6) and 1.8 (1.0-3.4) (not in Table). There was no significant difference between the groups with regard to distress caused

**Figure 1** Number and per cent of all men stating that they were severely distressed by a decrease in various aspects of sexual function. Severe distress refers to the highest category on a five-category ordinal scale. □, Men without prostate cancer; ■, men with prostate cancer.

**Figure 2** Number and per cent of all men stating that they were *severely* distressed by various urinary/bowel complications. Severe distress refers to the highest category on a four-category ordinal scale. □, Men without prostate cancer; ■, men with prostate cancer.

**Table V** Proportion of randomly selected men aged 50–80 stating their willingness to trade off the possibility of longer life expectancy against preserving their present level of sexual function (see question in Materials and methods section)

| | *All ages 50–80* | *Age 50–59* | *Age 60–69* | *Age 70–80* |
|---|---|---|---|---|
| Would not accept treatment | 56/299 (19%) | 10/60 (17%) | 18/108 (17%) | 28/129 (22%) |
| Would accept treatment only if it prolonged life | | | | |
| 10 years or more | 83/299 (28%) | 19/60 (32%) | 35/108 (32%) | 29/129 (22%) |
| 3–10 years | 36/299 (12%) | 4/60 (7%) | 10/108 (9%) | 22/129 (17%) |
| 1–2 years | 11/299 (4%) | 0/60 (0%) | 6/108 (6%) | 5/129 (4%) |
| Accept treatment unconditionally | 113/299 (38%) | 27/60 (45%) | 39/108 (36%) | 45/129 (35%) |

by other urinary or bowel symptoms. In an overall assessment of the effects of urinary and bowel symptoms, the proportion of all men reporting *severe* distress due to symptoms in one or more aspect of these functions was 4% (14/342) for the prostate cancer patients and 2% (6/314) for the men in the reference group (not in Table).

The prevalence of prostate cancer, diabetes mellitus and myocardial infarction was 2%, 6% and 11% in the reference group and 2%, 5% and 13% in the general population.

### Discussion

In the present study a decline in sexual desire, erectile and orgasm functions was the most common disease-specific distress in an unselected prostate cancer population. Sexual function as compared with their youth was diminshed in the majority of men in the investigated age group both with and without prostate cancer. The prevalence and severity of sexual dysfunction was significantly greater in the prostate cancer population. There was a large variation in the willingness to trade off increased survival for an intact sexual function, emphasising the need to tailor treatment decisions for each individual patient. Urinary and bowel symptoms were less prevalent than a waning sexual function and few men appeared to be severely distressed by them.

A decline in one or more aspects of sexual function was apparent in the majority of the investigated men. The prostate cancer patients were more likely to report a low frequency and intensity of erections compared with the men in the reference group. These findings were consistent with those of others (Schover, 1993; Litwin *et al.*, 1995). A waning sexual function distressed the majority of men with and without prostate cancer. For the men with prostate cancer our findings were corresponding to the previously published data (Pedersen *et al.*, 1993; Brasilis *et al.*, 1995; Helgason *et al.*, 1995; Litwin *et al.*, 1995).

The men were clearly discrepant in valuing their sexual function with approximately one-fifth not willing to risk their sexual function for any improvement in life expectancy, and another two-fifths not willing to trade off any possibility of a prolonged life for a preserved sexual function. This individual difference in willingness to risk the possibility of a longer life expectancy for an intact sexual function emphasises the heterogeneity in how men value their sexual function. However, a majority of the men stated they were willing to trade off life expectancy for an intact sexual function. In this respect our data for the men without prostate cancer correspond with the findings of Singer *et al.* (1991). The proportion of men with prostate cancer willing to do a trade-off was similar to the reference group but a comparison between the groups would be imprecise owing to a low number of surveyed patients with prostate cancer. Such data from men with prostate cancer has to our knowledge not been presented before.

The prevalence of urinary and bowel symptoms among the men with prostate cancer by and large correspond to data previously published (Perez *et al.*, 1986; Fowler *et al.*, 1993; Jönler *et al.*, 1995). However, few men in the present study appeared to be severely distressed by urinary and bowel symptoms. Of the investigated urinary symptoms, leakage was the most common cause of severe distress in both men with and without prostate cancer. Severe distress because of bowel symptoms was reported by only 1% of the patients and no men in the reference group. Of the men reporting any degree of urinary or bowel symptoms, the prostate cancer patients were significantly more likely to be severely distressed by urinary leakage, weak stream and bowel urgency.

Changes in sexual function, and to some extent urinary and bowel symptoms, reported by the men with prostate cancer, varied with the different treatments. However, a comparison of the various treatment groups was not the primary aim of the present study and comparisons are difficult to make as the patients were selected on clinical grounds to different treatments. For example, patients receiving endocrine therapy most likely had metastatic, locally advanced and/or poorly differentiated tumours. Patients subjected to radical surgery or radiotherapy only presumably had a clinically localised tumour. Men who had received endocrine therapy subsequent to radical surgery or radiotherapy most likely had tumours that had progressed after the initial treatment or had adverse prognostic findings at treatment. Those given no initial treatment probably had low-grade, clinically localised disease and were also older than the patients treated with curative intent. The initially untreated group may also have included some patients with metastatic or advanced disease without symptoms.

The response rate for the questionnaire was slightly lower for the reference group which may be owing to the fact that the majority of these men did not have any specific disease affecting the investigated functions, thereby making them less inclined to answer. The only significant difference between the reference group and the prostate cancer patients, with regard to reasons for not answering the questionnaire, was that the reference population was more difficult to locate (not living at registered address or living abroad) (Table I). We found no indication of a lack of representativeness at least not with regard to the prevalence of diabetes and myocardial infarction since the reference population was almost identical to the general Swedish male population in this respect.

The results show that waning sexual function is the most common disease-specific reason for distress in men with prostate cancer. Urinary and bowel symptoms are more prevalent in prostate cancer patients compared with the general population but few men are severely distressed by this. The importance of an intact sexual function for the patient and the willingness to abstain from treatment that may result in waning sexual function varies greatly between patients.

**Acknowledgements**

The authors are grateful to the Swedish Cancer Society and the Stockholm Cancer Foundation for financial support.

### References

BERGER C, ROCHER FP, ZHU Y, ROMESTAING P, AYZAC L AND GÉRARD JP. (1993). Activité sexuelle après irradiation pelvienne pour cancer de la prostate. *J. Urol. (Paris)*, **99**, 219–227.

BRASILIS KG, SANTA-CRUZ C, BRICKMAN AL AND SOLOWAY MS. (1995). Quality of life 12 months after radical prostatectomy. *Br. J. Urol.*, **75**, 48–53.

FOWLER FJ, BARRY MJ, LU-YAU G, ROMAN A, WASSON J AND WENNBERG JF. (1993). Patient-reported complications and follow-up treatment after radical prostectomy. *Urology*, **42**, 622–629.

HELGASON AR, FREDRIKSSON M, ADOLFSSON J AND STEINECK G. (1995). Decreased sexual capacity after radiation therapy for prostate cancer impairs quality of life. *Int. J. Radiat. Oncol. Biol. Phys.*, **32**, 33–39.

JÖNLER M, MESSING EM, RHODES PR AND BRUSEWICK RC. (1994). Sequelae of radical prostatectomy. *Br. J. Urol.*, **74**, 352–358.

LITWIN MS, HAYS RD, FINK A, GANZ PA, LEAKE B, , LEACH GE AND BROOK RH. (1995). Quality of life outcomes in men treated for localized prostate cancer. *JAMA*, **273**, 129–135.

PEDERSEN KV, CARLSSON P, RAHMQUIST M AND VARENHORST E. (1993). Quality of life after radical prostatectomy for carcinoma of the prostate. *Eur. Urol.*, **24**, 7–11.

PEREZ CA, PILEPICH MV AND ZIVNUSKA F. (1986). Tumour control in definitive irradiation of localized carcinoma of the prostate. *Int. J. Radiat. Oncol. Biol. Phys.*, **12**, 523–531.

ROTHMAN KJ. (1986). *Modern Epidemiology*. Little, Brown and Co.: Boston/Toronto.

SCHOVER LR. (1993). Sexual rehabilitation after treatment for prostate cancer. *Cancer*, **71**, 1024–1030.

SINGER PA, TASCH ES, STOCKING C, RUBIN S, SIEGLER M AND WEICHELBAUM R. (1991). Sex or survival: trade-offs between quality and quantity of life. *J. Clin. Oncol.*, **9**, 328–334.

STATISTICS SWEDEN. (1996). *The Swedish survey of living conditions*, Appendix 16. Statistics Sweden: Stockholm.